# The foot and ankle structures reveal emergent properties analogous to passive springs during human walking

**DOI:** 10.1371/journal.pone.0218047

**Published:** 2019-06-07

**Authors:** Erica A. Hedrick, Steven J. Stanhope, Kota Z. Takahashi

**Affiliations:** 1 Department of Biomechanics, University of Nebraska at Omaha, Omaha, NE, United States of America; 2 Department of Kinesiology & Applied Physiology, University of Delaware, Newark, DE, United States of America; University of Memphis, UNITED STATES

## Abstract

An objective understanding of human foot and ankle function can drive innovations of bio-inspired wearable devices. Specifically, knowledge regarding how mechanical force and work are produced within the human foot-ankle structures can help determine what type of materials or components are required to engineer devices. In this study, we characterized the combined functions of the foot and ankle structures during walking by synthesizing the total force, displacement, and work profiles from structures distal to the shank. Eleven healthy adults walked at four scaled speeds. We quantified the ground reaction force and center-of-pressure displacement in the shank’s coordinate system during stance phase and the total mechanical work done by these structures. This comprehensive analysis revealed emergent properties of foot-ankle structures that are analogous to passive springs: these structures compressed and recoiled along the longitudinal axis of the shank, and performed near zero or negative net mechanical work across a range of walking speeds. Moreover, the subject-to-subject variability in peak force, total displacement, and work were well explained by three simple factors: body height, mass, and walking speed. We created a regression-based model of stance phase mechanics that can inform the design and customization of wearable devices that may have biomimetic or non-biomimetic structures.

## Introduction

An objective understanding of human foot and ankle function can help identify the mechanisms that underlie healthy locomotion, and drive innovations and development of bioinspired wearable devices, such as prostheses and exoskeletons. For example, knowledge of the mechanical forces and work in the human foot and ankle are informative for understanding how the anatomical structures change the body’s energy states. Muscles in the lower limb such as the ankle plantarflexors do work through active muscles contractions [1–7]. Elastic structures such as the plantar fascia [8–11] and Achilles tendon [6, 7, 10, 12–16] can store and return mechanical energy [17]. Examining the work production of biological structures can determine what material characteristics (e.g., elastic or viscous) or components (e.g., battery-powered actuators) are needed for device designs to emulate natural structures and/or functions.

Bio-inspiration has been central to the recent development of ankle-related devices, such as exoskeletons and prostheses. The biological ankle structures, in particular the plantar flexor muscle-tendon structures, play an important role in providing body support, forward propulsion, and initiating swing [5, 13]. These structures produce the largest proportion of positive work during the stance phase of walking [2, 12, 18], utilizing elastic structures such as the Achilles tendon [6, 7, 10, 12–16], and active muscles [1–7]. As such, certain functions of the biological ankle have been emulated with unpowered and powered devices. For example, unpowered exoskeletons [19] or orthoses [20–22] designed with elastic materials can store energy through torsional stiffness at the ankle and provide push-off power via elastic energy return. Powered exoskeletons [23–27] and prostheses [28–30] emulate active plantarflexion by generating a burst of positive ankle power during late stance. These bio-inspired devices attempt to replicate how an isolated region (i.e., ankle joint) behaves in humans without necessarily precisely replicating the designs of these regions. However, this approach does not take into account the properties of more distal structures in the foot, which also exhibit unique energetic characteristics, including energy storage, return, [8–11] and/or dissipation [31–33]. We propose a more encompassing approach in designing ankle-foot devices, in which we examine the summed effect of how a group of structures act together during locomotion.

When all biological foot and ankle structures are combined, a recent study in walking found that the net effect of these structures resemble an energy neutral system producing near equal magnitudes of negative and positive work [34]. While the ankle plantar flexor muscle-tendon structures generate most of the positive work within the lower limb [1, 12, 34], foot structures dissipate energy, due to the heel pad [31–33] and the metatarsophalangeal joint [34–37]. In other words, the combined foot-ankle behavior can be analogous to a passive spring that stores and then returns energy (i.e., zero net work). This knowledge of the functions of biological structures could allow versatility in the design of wearable devices: the spring-like behavior of the biological foot-ankle system could be replicated, in theory, via elastic unpowered devices that store and return energy, or the function could be emulated via powered devices that can be actively controlled.

A generalized model that can parameterize the overall functions of the combined foot-ankle system is needed to further facilitate foot and ankle device designs and their customization. One important parameter will be the way in which the ground reaction force, or the location of the center-of-pressure, displaces during stance. It has been shown that the biological foot-ankle system conforms to a rocker-like shape during stance in walking, when the center-of-pressure displacement is viewed within the shank’s coordinate system [38–46]. Quantifying or parameterizing this ‘roll-over shape’ can be informative for understanding the overall behavior of the biological foot and ankle, and has also inspired wearable devices to preserve the biological ‘roll-over shape’ [41]. One example of recreating this ‘roll-over shape’ is using a solid ankle boot with a rocker bottom curvature [43]. In this example, the ‘roll-over shape’ is fixed and rigid, and effectively cannot perform mechanical work. The biological foot and ankle, in contrast, achieves its ‘roll-over shape’ through the combined effects of ankle joint and toe joint rotations, and plantar surface deformations of the foot [38]. Thus, device designs that mimic ‘roll-over shape’ may not replicate the mechanical work profiles of the biological foot and ankle. Incorporating forces, displacement and work profiles of the biological ankle and foot in a generalized model will lead to enhanced kinematic and work profiles of customized ankle and foot devices.

The purpose of this study was two fold. First, we aimed to quantify the combined force, displacement, and work of all structures distal to the shank (i.e., foot and ankle) during normal walking. Second, we tested the hypothesis that walking speed, body height, and mass are strong predictors for the individual variability of force, displacement, and work measures. This knowledge would facilitate creation of a simple data-driven, regression-based model that could predict idealized force, displacement, and work measures (distal to the shank) that may be used to customize unpowered wearable foot-ankle devices, such as prostheses and exoskeletons.

## Methods

### Experimental protocol

Eleven healthy subjects (6 females, 5 males, ages 24.2 ± 2.9 yrs, height 1.72 ± 0.08m, and body mass 75.3 ± 21.8 kg) participated in a fully-instrumented gait analysis. The subjects were screened for any musculoskeletal disorders, and gave written informed consent approved by the IRB at the University of Delaware. Kinematic data (120 Hz) were collected using a six camera-based motion capturing system (Eagle Cameras, Motion Analysis Corp., Santa Rosa, CA), and kinetic data (360 Hz) were collected from a strain gauge force platform (Model OR6-7-2000, 46.4cm x 50.8 cm, AMTI, Watertown, MA). A 6 degrees-of-freedom marker-set [47] was used to estimate lower extremity movement during walking. The subjects walked barefoot at four walking velocities: 0.4, 0.6, 0.8 and 1.0 statures/second (0.69 ± 0.03, 1.03 ± 0.05, 1.38 ± 0.06 and 1.72 ± 0.08 m/s), verified by two photocell beams located approximately 3.0 meters apart. Trials were accepted for analysis if the subject’s actual velocity was within ± 0.02 statures/second of the targeted velocity, and if the subjects’ entire right foot was observed to contact a single force platform. All data were processed and analyzed using Visual3D software (C-Motion Inc., Germantown, MD). A second-order low-pass Butterworth filter (6Hz for kinematic data, and 25Hz for kinetic data) was applied to the raw data.

### Force and displacement distal to the shank

**Fig 1:**
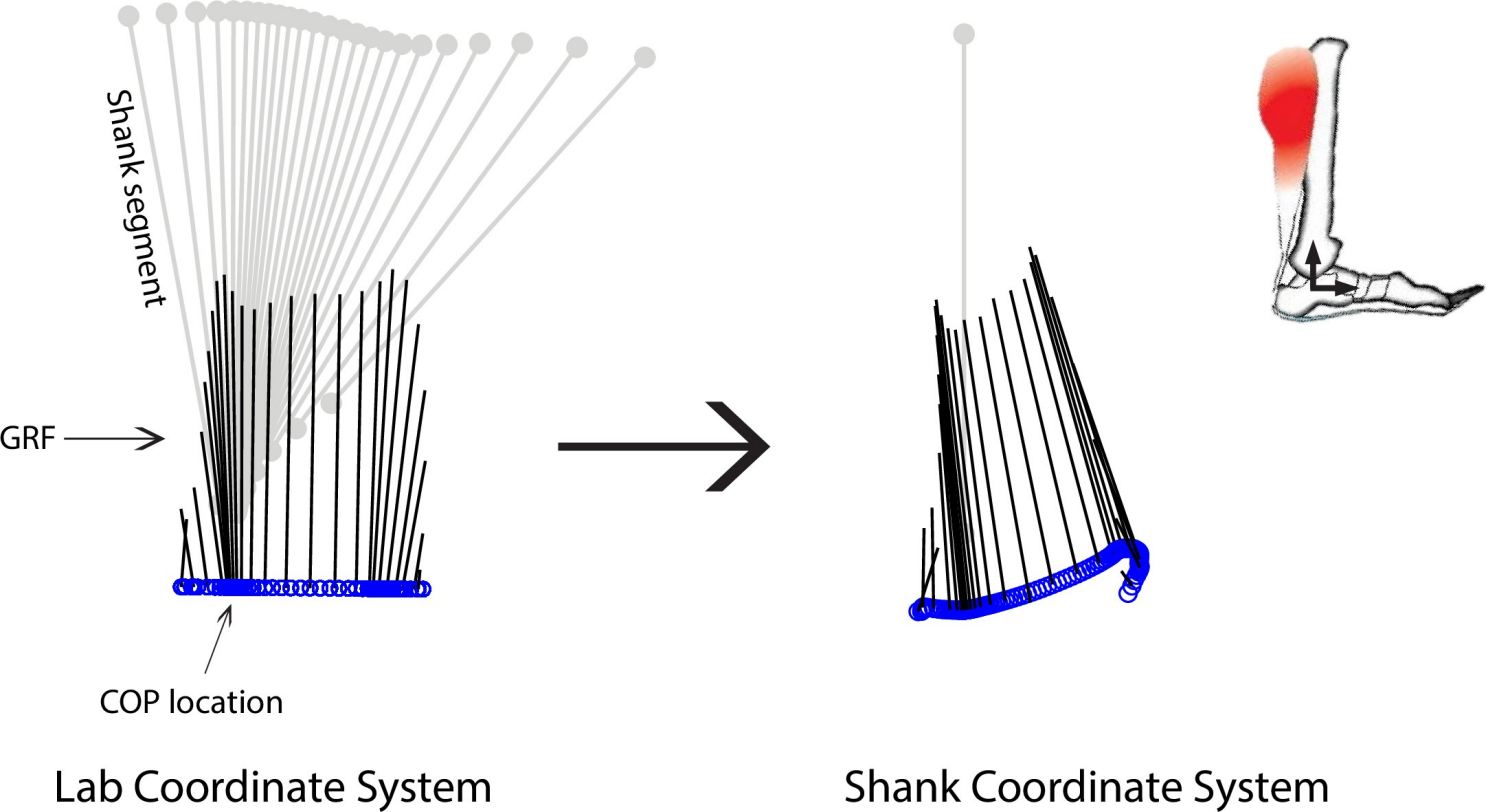
Coordinate transformation. Both the ground reaction force (GRF) and center of pressure (COP) data were transformed from the lab coordinate system into the shank coordinate system (SCS). This analysis characterized the force and displacement distal to the shank, respectively.

When center-of-pressure (COP) displacement trajectory (i.e., locations of ground reaction force (GRF) application during stance) is transformed from the laboratory coordinate system to the shank’s coordinate system (SCS), the COP displacement profile characterizes the ‘effective shape’ or the ‘net deformation’ of the ankle and foot structures [38, 41]. Using these concepts, we transformed both the GRF and COP data into the SCS to characterize the force and displacement distal to the shank, respectively (Fig 1). We established the SCS such that the X-axis defined the medial-lateral (M/L) axis, Y-axis defines the anterior-posterior (A/P) axis, the Z-axis defined the superior-inferior (S/I) axis, and the origin was represented by the ankle joint center. Altogether, these kinematic and kinetic data represented the force and displacement profiles of the ankle-foot structures.

### Power distal to the shank

We quantified the total mechanical power produced by all structures distal to the shank, using a unified deformable segment analysis [48]. This technique synthesizes the GRF and COP displacement relative to the shank, and captures the summed contributions of the ankle and foot structures [48]. Specifically, the COP velocity, or the ‘distal velocity’ ($\overset{⃑}{v_{d}}$), was quantified (Eq 1) by accounting for the center-of-mass (COM) translational velocity ($\overset{⃑}{v_{cm}}$) and segment rotational velocity $\left(\overset{⃑}{\omega_{s}}\right)$ of the shank, and movement of the COP displacement relative to shank (i.e., the vector from shank COM to COP $\left[{\overset{⃑}{r}}_{COP}\right]$).

$$\overset{⃑}{v_{d}}=\overset{⃑}{v_{cm}}+(\overset{⃑}{\omega_{s}}x\overset{⃑}{r_{cop}})$$ (1)

The term ‘distal’ here is used to signify the contributions of all structures distal to the shank, as estimates of $\overset{⃑}{v_{d}}$ can be influenced by ankle and/or foot mechanics. The estimate of $\overset{⃑}{r_{COP}}$, notably, is analogous to the COP displacement in the SCS (as defined previously). Then, the power of all structures distal to the shank (P) was quantified (Eq 2) by the summation of the dot product of GRF and $\overset{⃑}{v_{d}}$ and the dot product of force plate free moment (${\overset{⃑}{M}}_{free}$ – which is the moment about the vertical axis of the laboratory coordinate system) and ${\overset{⃑}{\omega }}_{s}$.

$$P=\overset{⃑}{GRF}∙\overset{⃑}{v_{d}}+\overset{⃑}{M_{free}}∙\overset{⃑}{\omega_{s}}$$ (2)

### Data analyses

The total COP displacement and the GRF in both the superior-inferior and anterior-posterior directions in the SCS were analyzed. Both of these values were calculated during stance phase, from heel strike to toe-off, with a minimum 20 N vertical GRF threshold. The total excursion of COP displacement in the anterior-posterior axis was determined from the difference between the minimum and maximum COP values. The total excursion of COP displacement in the superior-inferior axis was analyzed by subtracting the maximum COP value from the initial value at heel strike. The GRF peak in the superior-inferior direction was the second peak (during late stance), and the GRF peak in the anterior-posterior direction was the minimum GRF value in the anterior-posterior axis. The positive and negative work distal to the shank was determined by separately integrating the positive and negative power values over time, respectively, using MATLAB (MathWorks Inc., Natick, MA, USA). Net work was the sum of positive and negative work measures.

### Statistical analysis

A linear mixed-model ANOVA was used to determine the effects of body height, body mass and speed on the outcome variables (COP displacement, GRF peaks, positive work, negative work, and net work). The analysis was a mixed-model, four-factor ANOVA (random effect: subject; fixed effects: speeds, body height and body mass). All four factors were initially entered into the model, and stepwise elimination was used to eliminate the least significant variables until only the significant terms were left (p<0.05). The remaining significant variables were included in the predictor equation for the outcome variables. The coefficients for these variables, as well as the R^2^ value for the equation was reported. This analysis was done for each outcome variable (MATLAB; The MathWorks Inc., Natick, MA, USA and IBM SPSS Statistics, IBM, Armonk, NY, USA).

## Results

### Displacement

**Fig 2:**
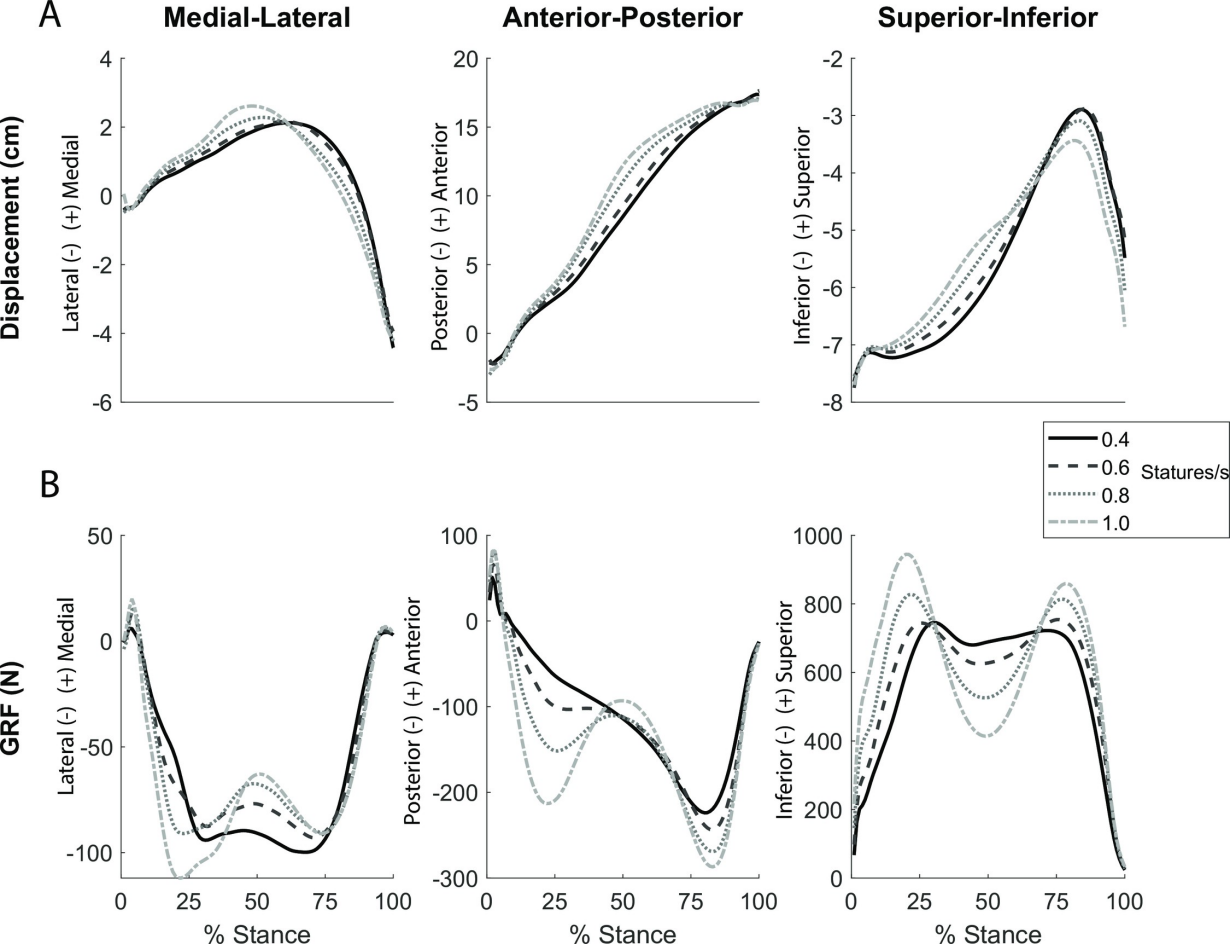
**Center-of-pressure displacement (a) and ground reaction force (b) time series in the shank coordinate system.** The different lines represent the 4 speeds (solid line = 0.4 statures/s, dashed line = 0.6 statures/s, dotted line = 0.8 statures/s, and dash dot line = 1.0 statures/s).

The COP in the SCS primarily displaced in the anterior direction during stance (Fig 2A). The total COP displacement in the anterior-posterior direction was 20.33 ± 1.68, 20.43 ± 1.45, 20.64 ± 1.56, and 20.41 ± 1.70 cm for each increasing speed (0.4, 0.6, 0.8, and 1.0 statures/s), respectively. According to the mixed model ANOVA, body height (p = 0.003) was a significant predictor of total displacement in the anterior-posterior direction (adjusted R^2^ = 0.76). Body mass and speed had a non-significant effect on A/P displacement, and thus weren’t included in the model. The model predicted the following equation: (Eq 3)

DisplacementA/P(cm)=−1.80+0.13*Height(cm) (3)

The COP displaced in the superior direction as the ankle dorsiflexed, and the COP displaced in the inferior direction as the ankle plantarflexed (Fig 2A). The total COP displacement in the superior direction was 4.80 ± 0.69, 4.93 ± 1.00, 4.52 ± 1.08, and 4.39 ± 0.91 cm for each speed (0.4, 0.6, 0.8, and 1.0 statures/s), respectively. The mixed model ANOVA determined that body height (p = 0.004) and speed (p = 0.0233) were significant predictors of total displacement in the superior-inferior direction (adjusted R^2^ = 0.71). Body mass had a non-significant effect and was therefore removed from the model. The resulting equation was: (Eq 4)

DisplacementS/I(cm)=−7.72−0.48*Speed(m/s)+0.08*Height(cm) (4)

#### Force

The ground reaction force in the SCS anterior-posterior axis was directed in the posterior direction for almost the entirety of stance, primarily because the shank predominately rotated in the forward direction around the medial-lateral axis during stance. The ground reaction force peak in the anterior-posterior (A/P) direction was -225.32 ± 52.86, -245.00 ± 56.24, -270.66 ± 62.16, and -292.03 ± 76.09 N for each increasing speeds (0.4, 0.6, 0.8, and 1.0 statures/s), respectively. Based on the mixed models ANOVA, body mass (p<0.001) and speed (p<0.001) were significant predictors of peak force in the anterior-posterior direction (adjusted R^2^ = 0.93). Body height had a non-significant effect on A/P peak force and was therefore removed from the model. The model equation became: (Eq 5)

A/PPeakForce(N)=−14.83–66.50*Speed(m/s)−2.17*Mass(kg) (5)

The overall pattern of the force in the superior-inferior axis was similar to the vertical GRF in the lab coordinate system [49] in that the force showed two distinct peaks during stance (Fig 2B). The vertical GRF peaks during late stance for each increasing speed were 728.44 ± 209.70, 758.16 ± 219.31, 815.60 ± 239.31, and 861.37 ± 272.52 N respectively. Body mass (p<0.001) and speed (p<0.001) were significant predictors of peak force in the superior-inferior direction (adjusted R^2^ = 0.98). Body height had a non-significant effect on S/I peak force and was therefore removed from the model. The resulting model equation is: (Eq 6)

S/IPeakForce(N)=−165.58+134.77*Speed(m/s)+10.54*Mass(kg) (6)

### Power and work

**Fig 3:**
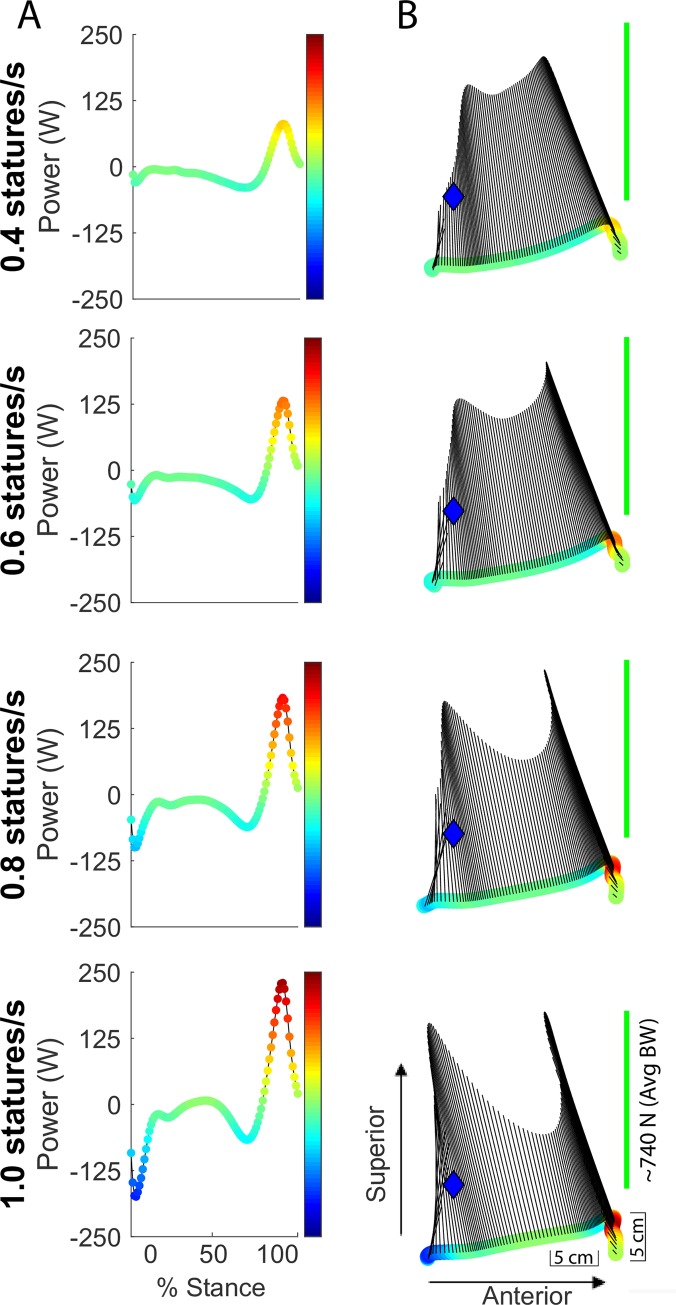
Displacement, force and power distal to the shank. (a) Average mechanical power distal to the shank during stance phase (N = 11). The colors correspond to the intensity of the instantaneous power. During the first ~75% of stance, the structures distal to the shank (i.e., foot-ankle) produced negative power, whereas during the last ~25% of stance, the structures produced positive power. (b) Displacement, force and power distal to the shank during stance phase. The colored circles indicate displacement of the COP in the shank coordinate system (SCS), and the colors of the circles represent power intensity at each point in stance. Black lines represent the ground reaction force in the SCS, in which the length of the vector denotes the magnitude of the force. Blue diamond represents the location of the ankle joint center in the SCS. The length of the green line represents the magnitude of the average body weight (~740 N).

**Fig 4:**
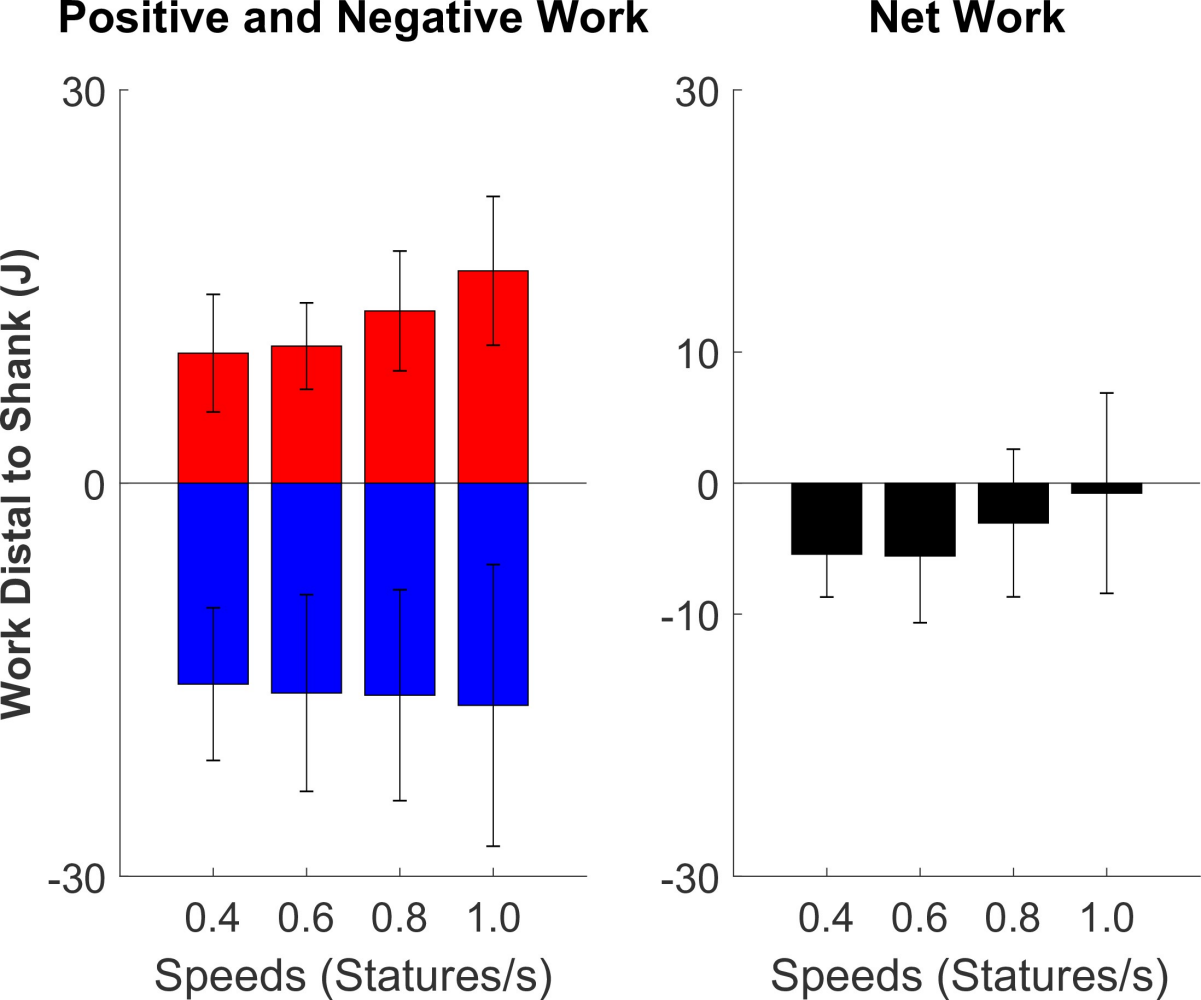
Average mechanical work distal to the shank during stance phase (N = 11). The magnitude of negative work (red) is slightly greater than positive work (blue), resulting in slight net negative work (black). The magnitude of positive work and net work increased with an increase in speed (p<0.001 and p = 0.0024), but not negative work (p = 0.1758).

During the first ~75% of stance, the structures distal to the shank (i.e., foot-ankle) produced negative power, whereas during the last ~25% of stance, the structures produced positive power (Fig 3). There was 9.92 ± 4.49, 10.46 ± 3.30, 13.14 ± 4.57, and 16.19 ± 5.68 J of positive work produced for the speed conditions of 0.4, 0.6, 0.8 and 1.0 statures/s, respectively (Fig 4).

The mixed model ANOVA revealed that body mass (p<0.001) and speed (p<0.001) were significant predictors of positive work (adjusted R^2^ = 0.86). Body height had a non-significant effect on positive work and was removed from the model. The resulting model equation was (Eq 7):

PositiveWork(J)=−7.61+6.23*Speed(m/s)+0.17*Mass(kg) (7)

The amount of negative work for each speed condition was -15.34 ± 5.83, -16.02 ± 7.50, -16.18 ± 8.05, -16.96 ± 10.74 J, respectively. These structures produced slightly greater magnitude of negative work than positive, resulting in slight net negative work (Fig 4).

Mass (p<0.001) was found to be a significant predictor of negative work (adjusted R^2^ = 0.90). Body height and speed had a non-significant effect on negative work and was removed from the model. The resulting model equation for negative work was (Eq 8):

NegativeWork(J)=9.68−0.34*Mass(kg) (8)

The amount of net work produced for each speed condition was -5.42 ± 3.27, -5.56 ± 5.09, -3.05 ± 5.63, and -0.77 ± 7.65 J. Body mass (p<0.001) and speed (p = 0.0023) were found to be significant predictors of net work as well (adjusted R^2^ = 0.62). Body height had a non-significant effect on net work and was removed from the model. The resulting model equation for net work was (Eq 9):

NetWork(J)=3.83+4.61*Speed(m/s)−0.17*Mass(kg) (9)

## Discussion

The purpose of this study was to quantify the total force, displacement, and work of all structures distal to the shank in normal walking. Quantifying the force and displacement aids in determining how the biological foot and ankle structures react to the dynamic loads, as well as determining the mechanical work performed by these structures. The distal to shank peak forces, total displacement, and work profiles can be used to create a data-driven model to help customize wearable foot-ankle devices. Our hypothesis was partially supported in that the subject’s speed, body height and mass did predict individual variability of peak force, total displacement and work. However, not all of the variables predicted force, displacement, and work.

In previous studies of the ‘roll-over shape,’ it was found that the COP displacement conforms to a circular shape from heel strike to opposite foot contact [38, 40–46]. Furthermore, this roll-over shape is influenced by the subject’s height and is unaffected by walking speed [38]. Our results are in partial agreement in that body height significantly predicted total COP displacement in both the superior-inferior and the anterior-posterior axes. However, we found that speed had an effect on COP displacement in the superior-inferior axes, but not the anterior-posterior axes. In this study, we analyzed each axes of the COP displacement separately, instead of looking at the radii of the curvature [38, 40–42, 45], which could have led to the differing results. We quantified the two axes separately so that we could relate the forces that would cause the displacement along each axes.

By analyzing the force and work, in conjunction with the center-of-pressure displacement, our analyses revealed an emergent property of human foot and ankle function that is analogous to a passive system. In agreement with prior studies [34], the foot and ankle performed net work that did not exceed zero (or was not positive) across various walking speeds, with the amount of negative net work increasing with slower walking speeds. During the slower walking speeds of 0.4 and 0.6 statures/s, the net work was 35.35% and 34.71% of the total negative work, respectively, suggesting that the foot and ankle structures behaved like a spring-damper system. However, as the speed increased to normal walking speeds of 0.8 to 1.0 statures/s, the net work became slightly negative and approaches near zero, while amounting to a much smaller percentage of the total negative work (18.84% and 4.51%). This indicates that the mechanical work production was similar to an ideal spring. Moreover, the spring-like behavior was further evident from the force and displacement profiles along the longitudinal axis of the shank. During the first phase, the COP displaced in the superior direction in the presence of a superior-directed force through most of stance. This may be analogous to a spring being compressed longitudinally, and coincided with the foot-ankle structures performing negative work (Fig 3). During terminal stance, the COP displaced in the inferior direction in the presence of a superior-directed force. This can be compared to a spring recoiling after being compressed, which coincided with the foot-ankle structures performing positive work.

Spring-like analogies of biological limbs, similar to what we found for the foot-ankle system, are ubiquitous in human locomotion studies. For example, the spring-mass model has been used to characterize the center-of-mass movement during the stance phase of running [50–53]. A torsional spring model has been used to describe the moment-angle relationship of the human ankle during stance phase of walking [39, 54–57]. An important characteristic of biological springs is the stiffness (or ‘quasi-stiffness’), as measured by the ratio of the peak force and displacement of the leg during running [51, 58–60], or by the ratio of the ankle joint moment and angular displacement during walking [39, 54, 55, 57, 61]. In line with these concepts, we quantified the longitudinal stiffness of the distal-to-shank structures, by computing the ratio of the peak force and total displacement (in the shank’s coordinate system). We found that the longitudinal stiffness increased with faster speed (p = 0.002), where the stiffness across the four speeds (0.4, 0.6, 0.8, and 1.0 statures/s) was: 153.48 ± 43.81, 156.22 ± 40.31, 187.27 ± 58.52, and 200.71 ± 58.83 N/cm. Our findings support the idea that humans can modulate stiffness of the lower extremity structures across a wide range of locomotor tasks, similar to how the entire leg can modulate stiffness when running on various surfaces [58], or how the ankle joint can modulate stiffness when walking faster [57, 61] or with added mass [57].

Near zero net work done by the human foot and ankle structures may suggest that, in theory, an unpowered device could emulate biological function, either as devices that work with the underlying anatomy (i.e., exoskeletons or orthoses) or replace the structures (i.e., prostheses). In recent years, advancement in unpowered device designs have shown great promise in either augmenting and/or restoring normal walking. A spring-loaded ankle exoskeleton, for example, could reduce metabolic cost of walking in healthy adults [62]. An unpowered prosthesis that harvests collision energy during heel strike could enhance push-off in individuals with limb amputation [63]. Notably, the performance of unpowered devices appear to be largely affected by the selection of key design parameters, such as stiffness. A device that is not too stiff, or too compliant, appears to be favorable for maximizing gait outcomes, as there are mechanical and energetic consequences of using a non-ideal stiffness [64–69]. Furthermore, another feature that may be important for unpowered devices is the ability to change its stiffness depending on the walking task [70]. As our study, and other studies have found [57, 58, 61], the biological limbs have an inherent ability to modulate joint/limb stiffness based on the demands of locomotion. As such, a device that incorporates micro-motors to alter the stiffness characteristics [70, 71] may be a viable solution to replicate salient features of the biological foot and ankle system.

**Fig 5:**
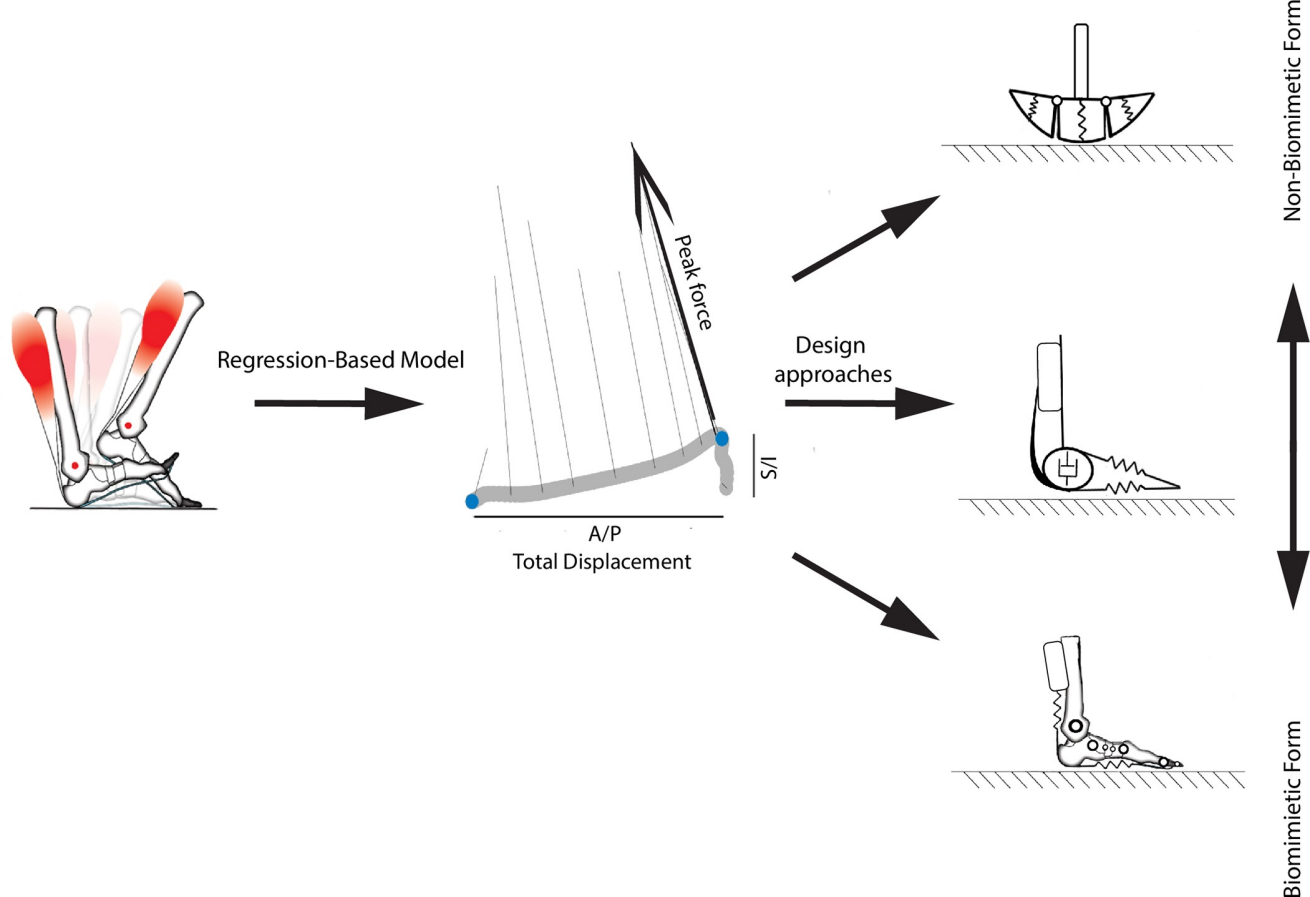
Data-driven regression model of the human foot and ankle function during walking. In particular, this model can predict an individual’s peak force and total displacement along the axes of the shank, as well as positive, negative and net work, based on body height, body mass and speed. This information may be translated into the design of prostheses, orthoses, or exoskeletons that attempt to emulate biological functions with a variety of designs, including both biomimetic and non-biomimetic approaches.

We developed a regression-based model of the biological foot and ankle mechanics (Table 1), and we envision that this model may be valuable for future design and customization of wearable devices. Most notably, we found that the subject-to-subject variability in peak force, total displacement, and work were well accounted for by three simple factors: the individuals’ body height, mass, and walking speed. This knowledge could then be translated into optimizing device characteristics for an individual user by customizing appropriate stiffness, shape, and/or damping (Fig 5). For example, for an individual who is 74.5 kg and 184 cm tall, with a desired walking speed at 1.3 m/s, our regression-based model would predict the following: total A/P displacement of 22.12 cm, total S/I displacement of 6.38 cm, A/P peak force of 262.95 N, S/I peak force of 794.85 N, positive work of 13.15 J, and negative work of -15.85 J. These parameters would enable a bio-inspired device to be designed that would replicate the function of the biological structures.

**Table 1:** Results of mixed-model ANOVA.

Variables	Intercept Coefficients	Height (cm)	Mass (kg)	Speed (m/s)
A/P Displacement (cm)	-1.80	0.13**	NA	NA
S/I Displacement (cm)	-7.72	0.08**	NA	-0.48*
A/P Force (N)	-14.83	NA	-2.17**	-66.50**
S/I Force (N)	-165.58**	NA	10.54**	134.77**
Positive Work (J)	-7.61**	NA	0.17**	6.23**
Negative Work (J)	9.68**	NA	-0.34**	NA
Net Work (J)	3.83	NA	-0.17**	4.61**

Results of mixed-model ANOVA (random effect: subject; fixed effects: subject height, subject mass, and speed; outcome parameters: A/P displacement, S/I displacement, A/P force, S/I force, positive work, negative work, and net work.

*P ≤ 0.05

**P ≤ 0.01

NA = not applicable to final model due to non-significant contribution.

There are some limitations of our model predictions. First, there are other mechanical (e.g., foot anthropometrics, muscle size) and neural (e.g., muscle activation) factors that could influence the peak force, total displacement, and work measures. Second, it is currently unclear how generalizable our model predictions are, since we did not test the accuracy of the model when applied to new individuals, either other healthy adults or persons with gait-related impairments. However, the advantage of our regression-based model is that it does not require biomechanics or gait-based data, and thus may offer a valuable ‘initial approximation’ towards a subject-specific device prescription. Our model then could be used in conjunction with existing ankle-foot devices that allow fine-adjustments in mechanical characteristics, such as prototypes that contain interchangeable components [62, 64, 65, 69] or through rapid-prototyping or 3-D printing technology [72–75].

By examining mechanics distal to the shank, designs of future foot and ankle devices need not be constrained to mimic biological form (Fig 5). While biomimicry has been central to many foot-ankle devices [28, 62, 76, 77], our generalized model of biological foot and ankle function is not restricted to structures that have anatomical resemblance, such as devices that have an ankle articulation of foot skeleton-like segments. In theory, replicating the ‘distal to shank’ force, displacement, and work profiles could arise from devices that take on non-biomimetic form. Such non-biomimetic devices are existent in robotic legs inspired from animals [78–81] and in many prosthetic ankle-foot devices that do not have true joint articulations [82–86], for example, running-specific prostheses that emulate spring-like behavior the human leg [83–86]. Our data-driven model may then promote versatility and flexibility in future designs that attempt to replicate biological function via biomimetic and/or non-biomimetic form.

### Conclusion

By examining the force, displacement, and work output from structures distal to the shank, we gained novel insights regarding human foot and ankle functions during walking. In particular, the foot and ankle system is analogous to an ideal spring that compresses and recoils along the longitudinal axis of the shank, performing near zero or negative net work across a range of walking speeds. The subject-to-subject variability in peak force, total displacement and work are predicted by a combination of body mass, body height and walking speed. These findings can create a database of normal gait that can inform the design and customization of unpowered foot-ankle device (e.g. prostheses, exoskeletons) that can emulate biological functions during walking.

## Supporting information

S1 Table Subject specific data and average data. Subject specific data contributing to the mixed-model ANOVA are included. The data also include average data contributing to the center-of-pressure displacement and ground reaction force, time series in the shank coordinate system, as well as the average mechanical power and work distal to the shank during the stance phase.
(XLSX)
